# Vertical crustal motions across Eastern Tibet revealed by topography-dependent seismic tomography

**DOI:** 10.1038/s41598-017-03578-z

**Published:** 2017-06-12

**Authors:** Xinyan Zhang, Yanghua Wang, Rui Gao, Tao Xu, Zhiming Bai, Xiaobo Tian, Qiusheng Li

**Affiliations:** 1 MLR Key Laboratory of Earthprobe and Geodynamics, Institute of Geology, Chinese Academy of Geological Sciences, Beijing, 100037 China; 2 Department of Earth Science and Engineering, Imperial College, London, SW7 2BP UK; 3 School of Earth Science and Engineering, Sun Yat-sen University, Guangzhou, 510275 China; 4 State Key Laboratory of Lithospheric Evolution, Institute of Geology and Geophysics, Chinese Academy of Sciences, Beijing, 100029 China

## Abstract

Using a topography-dependent tomographic scheme, the seismic velocity structure of the Eastern Tibetan Plateau, including the uplifted Longmenshan (LMS) orogenic belt, is accurately imaged in spite of the extreme topographic relief in the LMS region and thick sedimentary covers in the neighbouring Sichuan Basin. The obtained image shows a high-resolution upper crustal structure on a 500 km-long profile that is perpendicular to the LMS. The image clearly shows that the crystalline basement was uplifted within the LMS orogenic belt, and that the neighbouring Songpan-Ganzi Terrane was covered by a thick flysch belt, with evidence of near-surface thrust faults caused by convergence between Eastern Tibet and the Sichuan Basin. The indication that the lower crust beneath the LMS was folded and pushed upwards and the upper crust was removed by exhumation, supports the concept of a lower crustal channel flow beneath Eastern Tibet. The image also reveals that the destructive Wenchuan earthquake of year 2008 occurred in the upper crust, directly at the structural discontinuity between Eastern Tibet Plateau and the Sichuan Basin.

## Introduction

**Figure 1:**
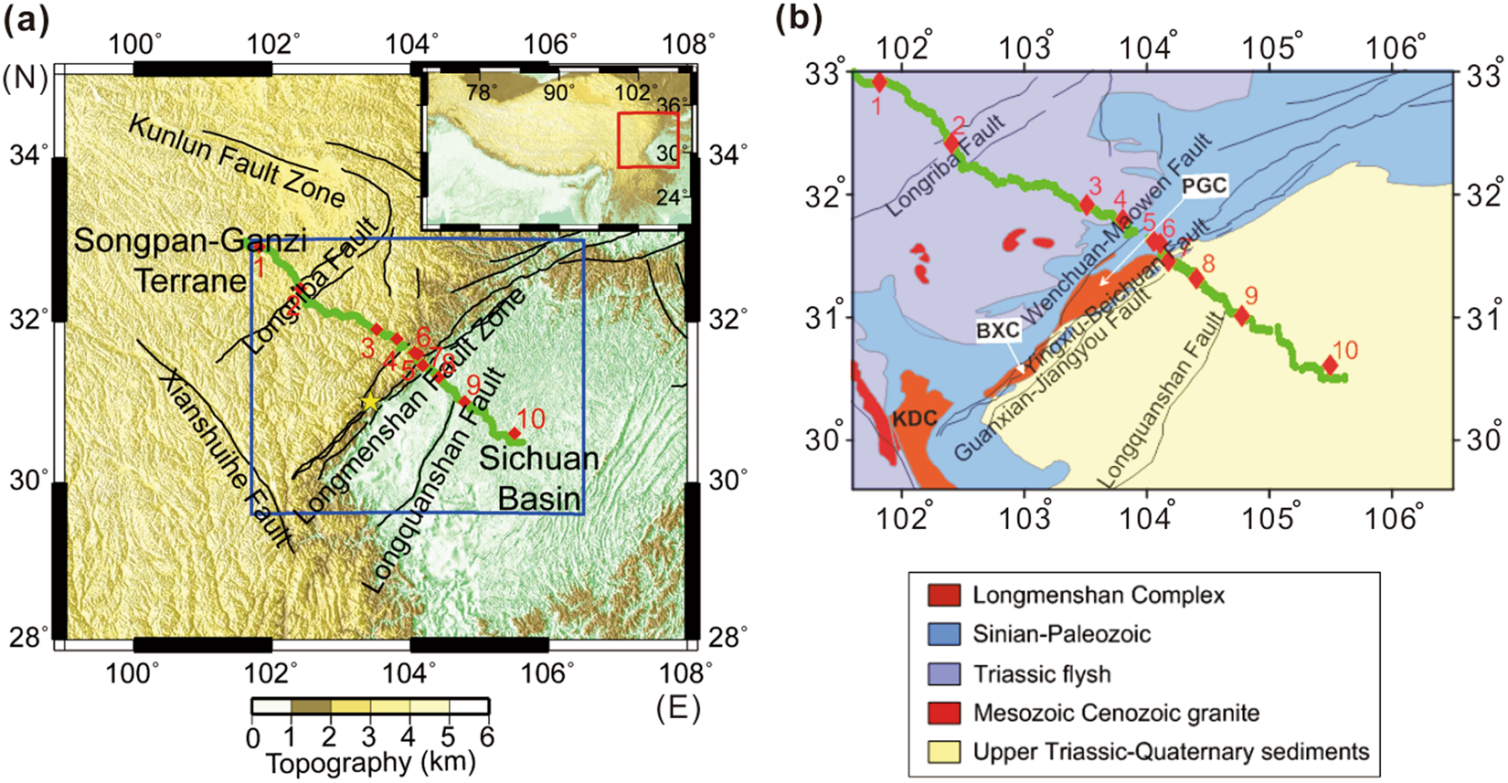
Tectonic and geological maps. (**a**) Tectonic map of the deep seismic sounding profile in the Eastern Tibet. Red diamonds denote shot positions (number 1, 2, …, 10), and green points denote receiver positions. Yellow star is the epicentre of Wenchuan earthquake. (**b**) Geological map to show the study area. Three Precambrian metamorphic complexes from the northeast to southwest are Pengguan complex (PGC), Baoxing complex (BXC) and Kangding complex (KDC). The blue rectangular box in (**a**) indicates the range of the geological map shown in (**b**). Maps are created using GMT (Generic Mapping Tools, http://gmt.soest.hawaii.edu/) software.

The collision between the Indian subcontinent and Asia in the late Eocene resulted in crustal thickening and the high elevation of the Tibetan Plateau^1–7^. The eastward moving crust of the Tibetan Plateau was obstructed at the Longmenshan (LMS) orogenic belt by the rigid Sichuan Basin^8, 9^. Thus, the LMS orogenic belt (Fig. 1) is the eastern margin of the Tibetan Plateau, and was uplifted by the extrusion of rigid crustal blocks along the left lateral strike-slip faults^10^.

This orogenic belt is characterised by a steep topographic gradient, as the elevation rises from ~500 m on the eastern part (the Sichuan Basin) to over 4 km in height on the western part (in Eastern Tibet) within a short horizontal distance of less than 50 km^11, 12^. It has been speculated that the build-up of this orogenic belt experienced a two-phase growth during the Cenozoic^5, 12–16^ and was a result of the eastward flow of the deep crustal viscous channel within Eastern Tibet^5, 14–18^ or lithospheric thrust faulting with large amounts of slip^19–21^. Here, we present a high-resolution image of the upper crustal velocity structure that enables us to accurately evaluate these interpretations.

In this paper, using seismic refraction tomography results, we re-interpret a 500-km long deep seismic sounding (DSS) profile that traverses the LMS orogenic belt in a perpendicular manner^22^. In the tomographic inversion for reconstructing a velocity model with such a steep topographic relief, we adopted a topography-dependent scheme^23^ which generates a uniform ray path distribution in the imaging area and, therefore, has a well-constrained upper crustal velocity model. Using this velocity model for geodynamic interpretation, we are able to understand not only how the Tibetan Plateau interacted with the Sichuan Basin locally at the LMS orogenic belt but we also, potentially, able to understand the tectonic mechanism of large earthquakes which occurred beneath the orogenic belt.

The LMS orogenic belt is a part of the northeast-southwest trending seismic fault zone. The devastating Wenchuan earthquake (Ms 7.9, 12 May 2008) occurred right beneath the LMS orogenic belt^24, 25^. The two neighbouring regions of the LMS orogenic belt are the Songpan-Ganzi Terrane in the northwest, and the Sichuan Basin in the southeast. The former is a Triassic orogenic belt, and the latter is a stable craton covered by undeformed Mesozoic-Cenozoic sediments. Along the DSS profile from northwest to southeast, the Longriba Fault (in the interior of the Songpan-Ganzi Terrane), the LMS Fault Zone, and the Longquanshan Fault (in the Sichuan Basin) are distributed sequentially (Fig. 1a). Within the LMS orogenic belt, a distinguishing feature of the surface geology is the Precambrian metamorphic complexes which are widely distributed from the northeast to the southwest along the strike direction (Fig. 1b)^24, 25^. In this study, we imaged the two-dimensional (2-D) DSS section across the most north-eastern complex, the Pengguan complex, which is assumed to have a high velocity in the near surface, relative to that in neighbouring regions.

Several DSS profiles have been previously studied for interpreting the upper crustal structure beneath Eastern Tibet and the Sichuan Basin^22, 26^. These studies approximated the abrupt topographic relief in the imaging process by simply filling in an artificial layer on the top to create a flat surface. This led to a lack of detail in the upper crustal models, as well as occasional inaccuracy^27^. Only by taking into account the strong topographic variation in the seismic tomography^28^ can we obtain a high-resolution velocity model and, therefore, make a reliable interpretation of the upper crustal structure.

## Data

**Figure 2:**
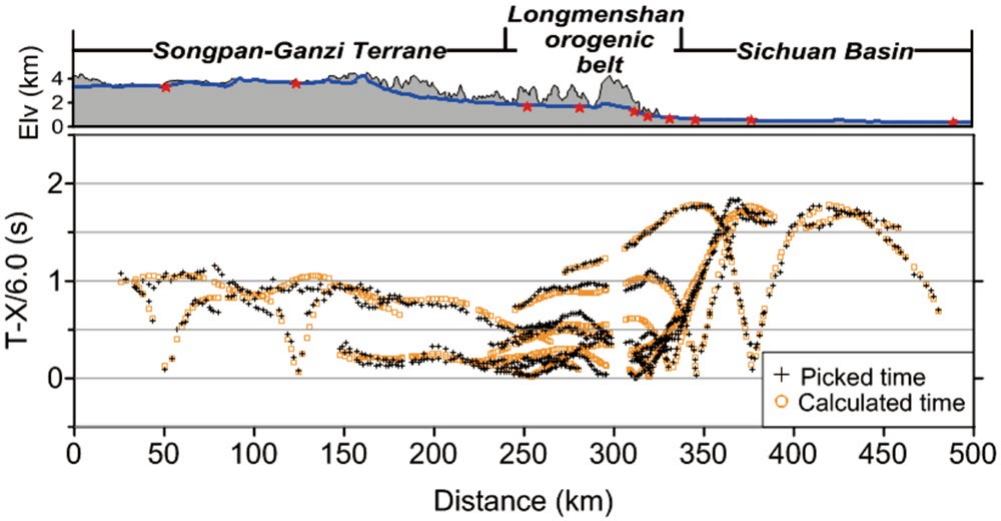
Distribution of first-arrival traveltimes. Black crosses denote traveltimes picked from the wide-angle seismic data and brown circles denote the final inverted traveltimes. These traveltimes are reduced by a velocity of 6 km/s. The top plate shows the elevation along the profile with the tectonic units labelled. The blue line and red stars denote receiver and shot positions, respectively.

Along the 500-km long DSS profile, 440 receivers (portable digital seismometers) were placed in order to record seismic data. These receivers were unevenly spaced at distance intervals within the range of 0.4 to 2 km (Fig. 1). Ten seismic shot records from the northwest to the southeast were selected for the tomographic analysis. They were spaced at distance intervals within the range of 10 to 80 km and labelled Sp1, Sp2, …, Sp10 (Supplementary information). Within the LMS orogenic belt, shots and receivers were both placed more densely, enabling dense ray coverage beneath the target range. From these ten shot records, we picked a total of 569 first arrivals of refraction waves, propagated through the upper crust, within a maximum source-receiver offset of 140 km. Figure 2 depicts the traveltime-distance relationship for these first arrivals as black crosses. The relationship is summarised in what follows.

First, in the Songpan-Ganzi Terrane (profile distance 0–230 km) in the northwest, the diving wave within the sedimentary cover, Psed, was clearly presented within a source-receiver offset of less than 20 km. The apparent velocity of Psed was about 4.0–5.3 km/s, and the reduced traveltime was 0–0.8 s. The diving wave within the crystalline basement, Pg, followed the Psed and was recorded at the offset 20–140 km. This long observable offset (~140 km) indicates that there is no low-velocity layer in the upper crust. The apparent velocity of the Pg was about 5.8–6.3 km/s and hence the reduced traveltime was 0.8–1.0 s. The diving wave turning in the upper mantle, Pn, was difficult to discern due to the low signal-noise ratio at a long source-receiver offset.

Second, in the Sichuan Basin (profile distance 330–500 km) in the southeast, Psed was observed at a source-receiver offset of up to 50 km, longer than the maximum offset in the Songpan-Ganzi Terrane. The apparent velocity of Psed was 3.5–5.2 km/s and the reduced traveltime was 0–1.8 s. The Pg was traceable from the offset of 50 km to 100 km. Although the apparent velocity of the Pg was roughly the same as that in the Songpan-Ganzi Terrane, the reduced traveltime was 1.5–1.8 s. This indicates the existence of a relatively thicker sediment layer, sitting on top of the crystalline basement.

Third, at the LMS orogenic belt in the structural transfer zone (profile distance 230–330 km), there was no Psed but a strong Pg was observed. The apparent velocity of the Pg was the same as that in the neighbouring regions; however, the reduced traveltime was the lowest, at 0–0.7 s. This corresponds well to the high velocity in the crystalline basement outcrops^25^.

In summary, these features of refraction waves clearly confirm the existence of the near-surface geological characteristics: a sedimentary cover in the Songpan-Ganzi Terrane, a high-velocity surface lithology and no sedimentary cover in the LMS orogenic belt, and thick sediment in the Sichuan Basin. These characteristics are confirmed by the high-resolution seismic tomography used in this study.

## Result

The elevation of sources and receivers along the 500-km long DSS profile is variable from 4.2 km to 0.3 km. To take account of this strong elevation variation in the upper crustal model reconstruction, we used a topography-dependent tomography scheme in which grids conform to the curved boundary.

In this study, because of the irregular topography, only pseudo-orthogonal grids were defined in the tomographic inversion^29^. These pseudo-orthogonal grids in the Cartesian coordinate were then transformed to a curvilinear coordinate. This transformation between two coordinates follows Poisson’s equation^30, 31^. The traveltime equation, i.e., the eikonal equation, was also transformed to the curvilinear coordinate for calculating the first-arrival traveltime field^23^. Given the traveltime field, traveltime gradients were computed spatially and ray paths were determined following the steepest gradient direction.

The inversion was carried out using a back-projection algorithm, in which traveltime residuals are uniformly projected along ray paths. The inversion procedure was stabilised by a smoothing operation, which controls the magnitude of the model update and spreads a time residual of a single ray path over a beam^32^. Synthetic traveltimes that were calculated from the inverted velocity model matched well with the traveltimes that were picked from seismic data (Fig. 2). The root-mean-square traveltime residual was finally reduced to 0.11 s.

**Figure 3:**
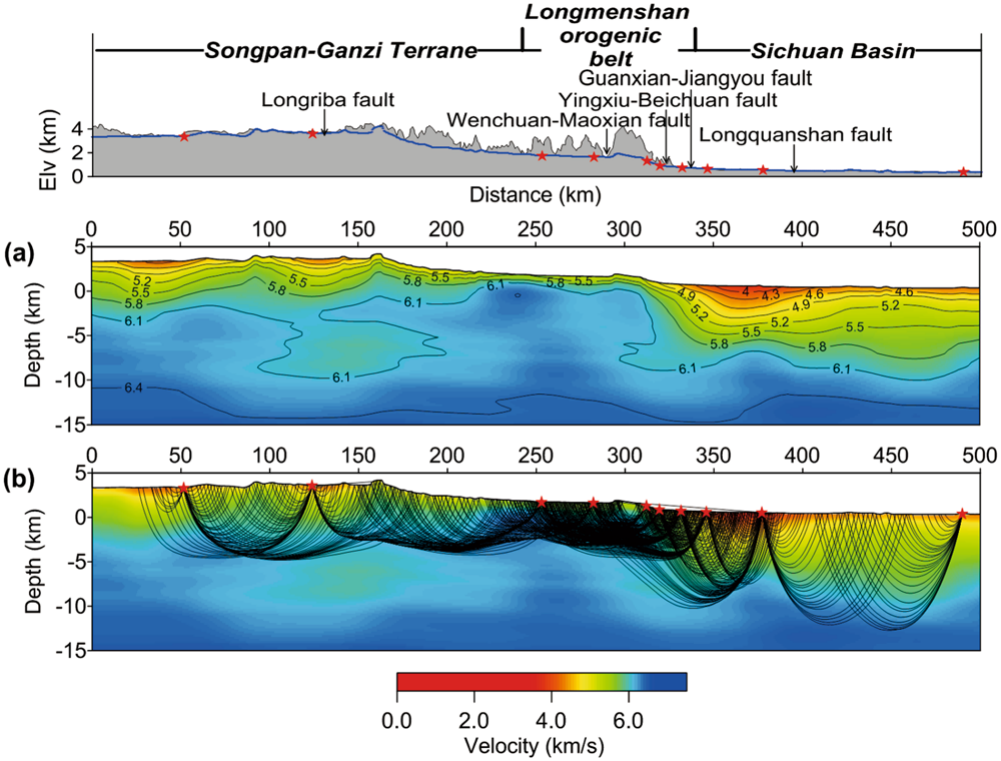
Upper crustal velocity model obtained from first arrival time tomography. (**a**) The velocity model obtained from tomography. (**b**) Ray paths penetrating through the upper crust.

Topography-dependent tomography produced a high-resolution upper crustal velocity model (Fig. 3a), which is well-constrained according to the illumination of ray paths penetrating through the media (Fig. 3b). Beneath the Songpan-Ganzi Terrane, seismic rays reach a thickness of 9 km below the surface (depth from 4 km to −5 km). Beneath the LMS orogenic belt, the penetration depth is much shallower (about 6 km thick, depth from +2 km to −4 km), demonstrating the high velocity of the upper crust. However, beneath the Sichuan Basin, seismic rays reach down to a thickness of 12–15 km (depth from +1 km to −14 km).

**Figure 4:**
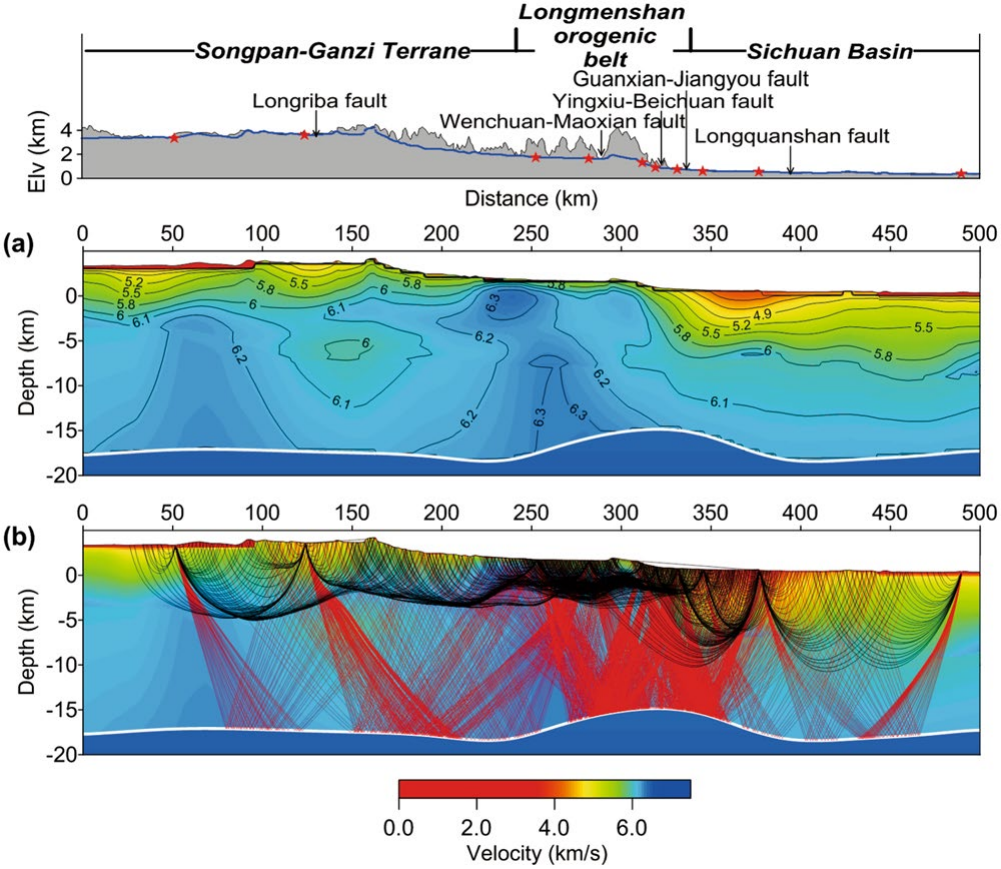
Upper crustal velocity model obtained from joint inversion of the first-arrival and reflection times. (**a**) Velocity image of the upper crust with an intra-crustal interface, at the average depth of 17 km. (**b**) Ray paths of the first arrivals and the reflection phase.

In order to verify this upper crustal structure model, a joint inversion of the first-arrival times and reflection times, reflected from the intra-crustal interface, was conducted (Supplementary information). Figure 4 is the joint-inversion result and the ray paths in the final model. The penetration depth of the reflection ray paths is about 17 km. This updated velocity model confirms that the high-velocity anomaly is reliable at a depth greater than 5 km beneath the Longmenshan faults. The sub-vertically continuous high-velocity column and the uplifted interface beneath Longmenshan orogenic belt may be caused by the lower crustal extrusion.

## Discussion

The high-resolution image of the upper crustal structure discloses significant features for each tectonic block: Thick sediments in the Sichuan Basin are juxtaposed against the thickened crust of Eastern Tibet, with compressional structure indicating the collision process.

**Figure 5:**
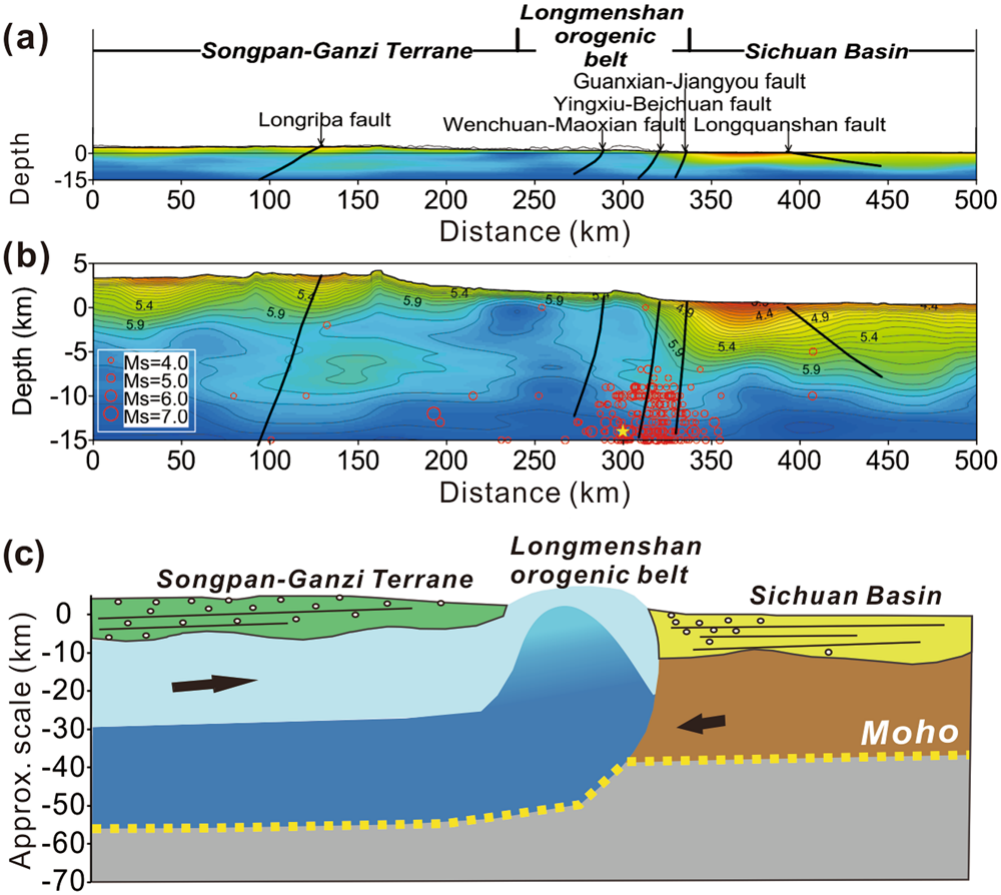
Velocity structure and tectonic model. (**a**) A series of steeply dipping faults with relatively true dips deduced from the fold structure near the surface. (**b**) The high-resolution velocity structure model, overlaid by deduced faults at depth and earthquakes. Red circles denote epicentres of the earthquakes that occurred along the profile since 1978, and the size of circles represents the magnitude. Yellow star is the epicentre of Wenchuan earthquake. (**c**) An interpretive tectonic model of Eastern Tibetan Plateau inferred from the upper crustal model. The arrows indicate the relative motion of the tectonic units. The high-velocity column beneath the LMS fault zone indicates the lower crustal expulsion which contributes to the LMS uplifting.

The velocity model is consistent with the geological survey along the profile^24, 33^. Figure 5a plots a series of steeply dipping faults with relatively true dips which are deduced from the fold structure near the surface. Figure 5b shows that lateral velocity anomalies distributed in the upper crust correspond well with faults observed on the surface.

The Triassic flysch sediment in the Songpan-Ganzi Terrane is clearly imaged in Fig. 5b, and has a thickness of 8–9 km at the west end. Although the thickness of this sedimentary cover decreases generally from west to east, the undulating bottom interface of the low-velocity layer may be the response of fold deformations, caused by the upper crustal thrust-sheet^34^.

The thick stratified sedimentary cover in the Sichuan Basin has a thickness of about 10 km, and shows a clear horizontal structure. An important feature of this sediment cover is the transpressive structure in the western Sichuan Basin between the Jiangyou-Dujiangyan fault and the Longquanshan fault^35, 36^. This transpressive structure is caused mainly by lateral expansion and the gravitational effect of the thrust-sheet LMS. It may also have be reformulated subsequently by denudation^37, 38^, which is enhanced by abundant rainfall and abrupt topographic relief.

In contrast to the two neighbouring regions, there is no sediment covering the LMS orogenic belt, where the near-surface velocity value is as high as 5.6 km/s. This is consistent with the Pengguan complex distribution on the surface, and indicates the uplifted crystalline basement and subsequent denudation.

In the uplifting process of the LMS thrust-sheet belt, fault dislocation occurred repeatedly, inducing a large number of earthquakes. In Fig. 5b, epicentres within ±0.5 degrees are projected to the upper crustal structure model (China Earthquake Data Centre). Beneath the eastern half of the LMS orogenic belt, there is a rapid velocity change in the horizontal direction. This rapid change corresponds well to the dense earthquake distribution within the belt.

Tectonic studies have postulated that crustal thickening caused eastward channel flow in mid- and lower crust of the Tibetan Plateau, which was obstructed by the lithosphere of the Sichuan Basin^14, 15, 37, 39^. This postulation has been verified by geophysical observations^40, 41^. Thermal and mechanical numerical models of deformation have demonstrated the upward expulsion of the Eastern Tibetan Plateau lower crust into the LMS orogenic belt with aggressive erosion^16, 17, 42^. Our high-resolution velocity structure (Figs 3 and 4) provides strong confirmation of these speculations and numerical simulations on the extrusion of the lower crust beneath the LMS orogenic belt. In this paper, we construct a tectonic model (Fig. 5c) in which we interpret the high-velocity column beneath the LMS orogenic belt as an indication of the lower crustal expulsion. This extrusion contributes, either additively or dominantly, to the uplifting of the LMS orogenic belt. We also interpret that the lower crustal folding due to the obstruction of the Sichuan Basin lithosphere was the prime cause of the destructive Wenchuan earthquake in 2008.

## Conclusions

Topography-dependent seismic tomography produced a high-resolution image of the upper crustal structure of the Eastern Tibetan Plateau. The image shows that the collision between the Tibetan Plateau and the Sichuan Basin compressed the upper crust along the profile, and that the fact of LMS orogenic belt being uplifted and tilted westwards led to the thickening of flysch sediment of the Songpan-Ganzi Terrane in a direction from southeast to northwest. Whereas the sedimentary cover in the Songpan-Ganzi Terrane was folded, the sediment of the Sichuan Basin clearly shows a transpressive structure. The lower crust was pushed upwards and exhumed by erosion at the LMS orogenic belt. This interpretation supports the concept of a lower crustal channel flow beneath Tibet. The image also indicates that the site of the destructive Wenchuan earthquake is directly at the upper crustal discontinuity between Eastern Tibet and the Sichuan Basin. Therefore, the high-resolution image of the upper crustal structure confirms the surface observations and geodynamic speculations of the Eastern Tibetan Plateau.

## Electronic supplementary material

Supplementary information
