# Supplementary material for: Vertical crustal motions across Eastern Tibet revealed by topography-dependent seismic tomography
Source: Sci Rep. 2017 Jun 12;7:3243. doi: 10.1038/s41598-017-03578-z (PMC5468289; doi:10.1038/s41598-017-03578-z)
Supplement: Supplementary file 1 — Supplementary information [file 41598_2017_3578_MOESM1_ESM.pdf]

Supplementary information

Vertical crustal motions across Eastern Tibet revealed by topography-dependent seismic tomography, by X. Zhang, Y. Wang, R. Gao, T. Xu, Z. Bai, X. Tian & Q. Li

S1. Detailed parameters of each shot record

Ten seismic shot records were selected for analysis. They were spaced at distance intervals in the range of 10 to 80 km and labelled Sp1, Sp2, …, Sp10 from the northwest to the southeast. Fig. S1 displays these ten shot records, marked with the first refraction phases.

The following table lists detailed coordinates and elevations of all ten shots:

Shot Number	Longitude (E)	Latitude (N)	Elevation (m)	Shot charge (kg)	Number of wells	Depth of well (m)
1	101°48.8226'	32°55.1377'	3468	2814	1	42.5
2	102°24.2320'	32°24.7443'	3596	2688	3	20.8
3	103°48.1650'	31°47.4255'	1712	600	2	26.5
4	104°03.2060'	31°35.8650'	1310	1000	1	10
5	104°10.3488'	31°27.6445'	688	600	1	35
6	104°18.4311'	31°23.2383'	486	800	2	20
7	104°24.2546'	31°18.9301'	530	1000	1	47.5
8	104°33.0524'	31°11.0742'	465	500	1	49
9	104°46.7510'	31°00.6347'	419	800	1	58
10	105°30.0357'	30°36.8452'	280	1800	1	61

Before the data acquisition, all seismometers underwent consistency verification to make sure that all of them could record a wide-angle event simultaneously. During the field experiment, all explosions and recorders were synchronised with GPS time. The sampling rate was 5 ms, and hence the time error between any seismometers is less than 5 ms.

S2. Resolution analysis of topography-dependent traveltime tomography

In this paper, traveltime tomography is carried out by using a back-projection algorithm. The initial velocity model is a 1-D function of depth, between depths 5 km and −15 km. The velocity is increased linearly from 4.0 to 6.5 km/s. The velocity model is updated via iteration. The final velocity model is the output after 19 iterations. Although the RMS traveltime residual is initially about 1.1 s, it has been decreased steadily to 0.09 s through the iterative inversion.

To confirm the main features of the tomographic result, we have conducted two kinds

of resolution analyses, for the topography-dependent traveltime tomography (TDTT) solver.

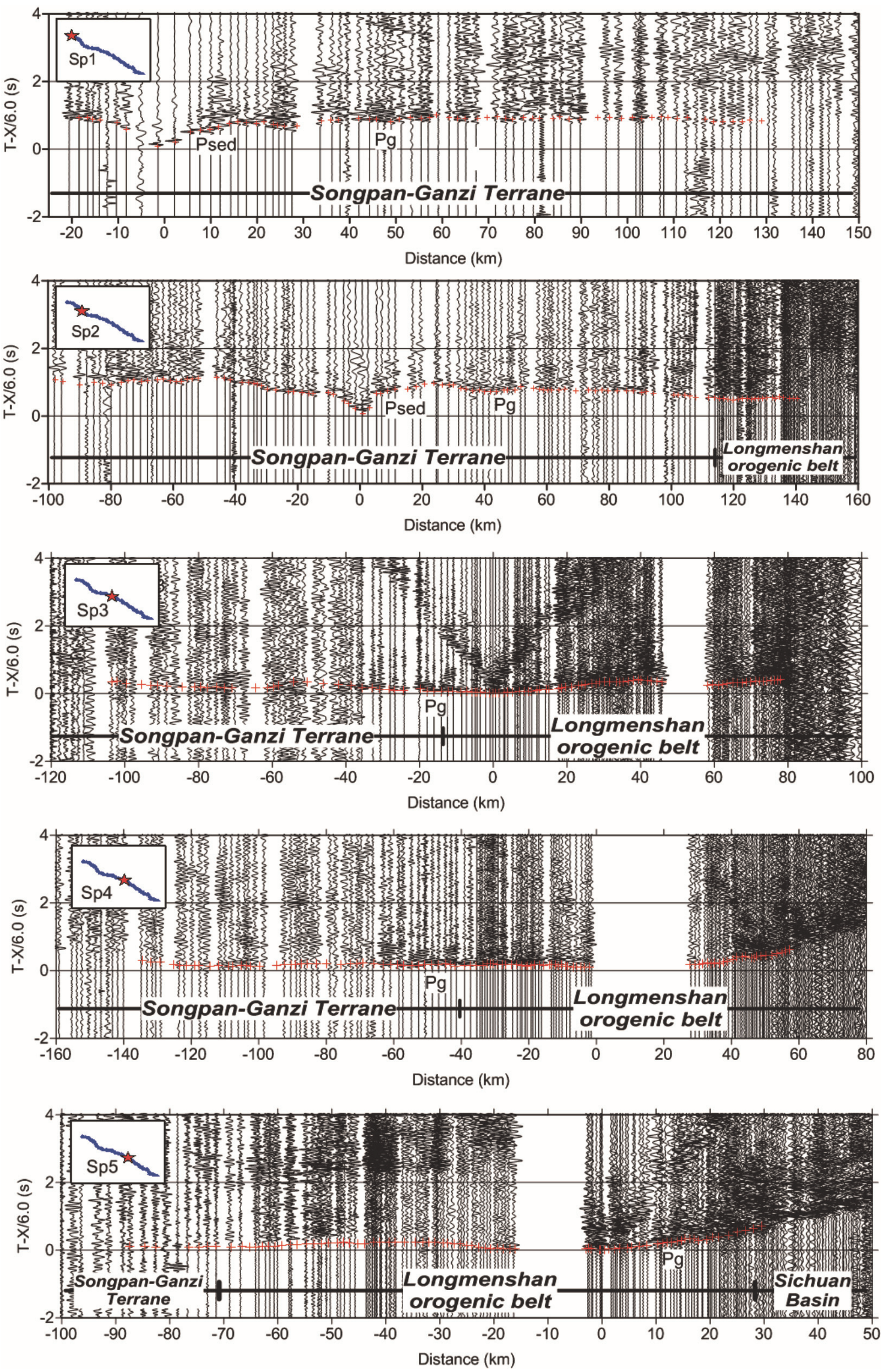

(*cont.*)

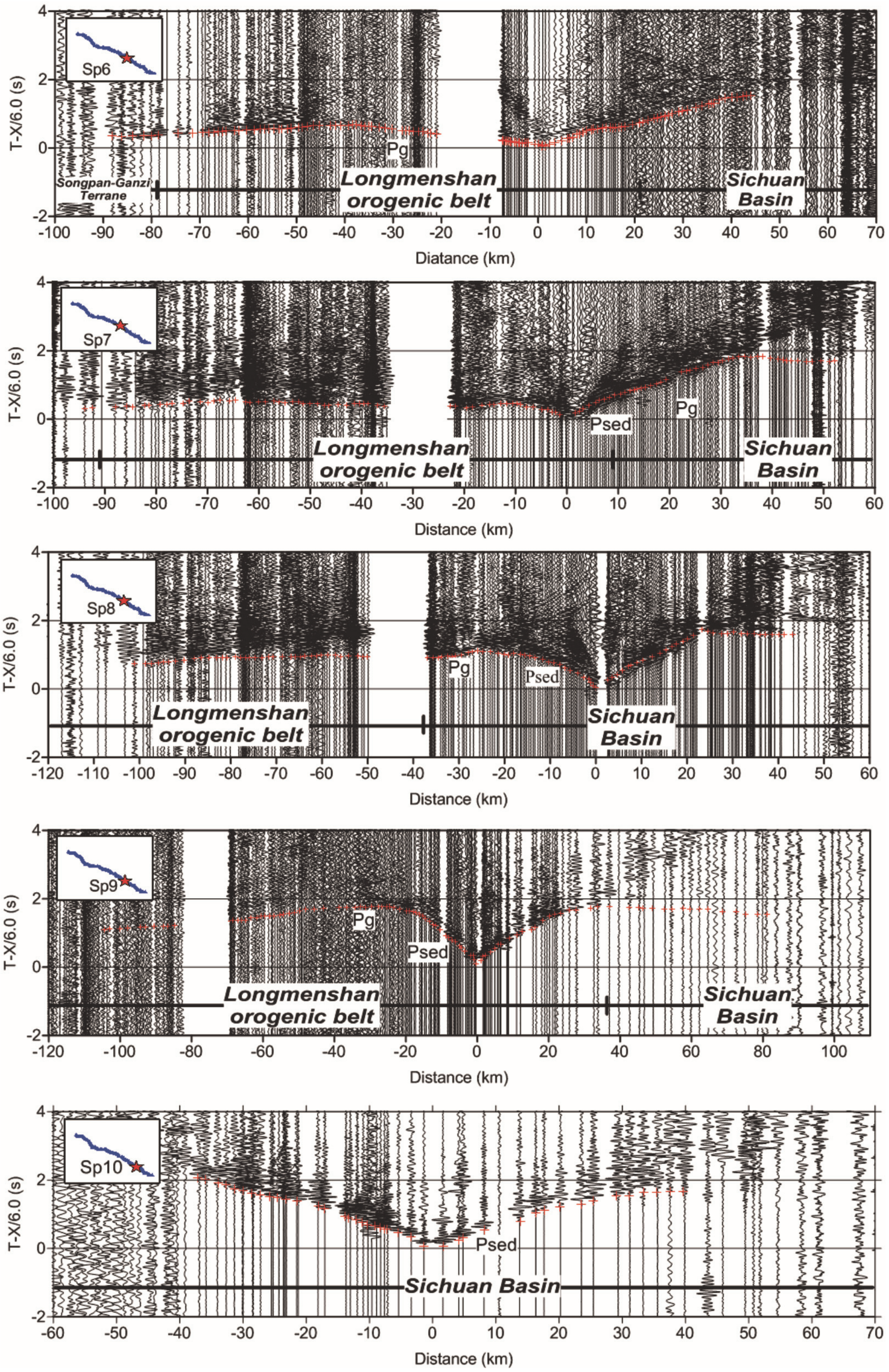

Figure S1. Ten shot records along the profile. Traces are normalised and bandpass-filtered (1–10 Hz). They are displayed with a reduced velocity of 6.0 km/s.

First, we apply a checkerboard test to evaluate the resolution as per similar observation surveys. We add alternate high and low velocity anomalies on the obtained tomographic velocity model. The velocity anomalies are described by formula $0.3 \times \sin x \times$

sin*z* (km/s), and with a space scale of 30 km (horizontal) × 15 km (vertical). When we invert the synthetic data, which are calculated from the checkerboard model, we apply the tomographic model (Fig.3a) as the initial model, and use the TDTT scheme. Comparison between the theoretical and recovered velocity anomalies, as shown in Fig.S2, indicates that the checkerboard pattern is well recovered along the wide-angle profile, which shows a high resolution of the data and scheme.

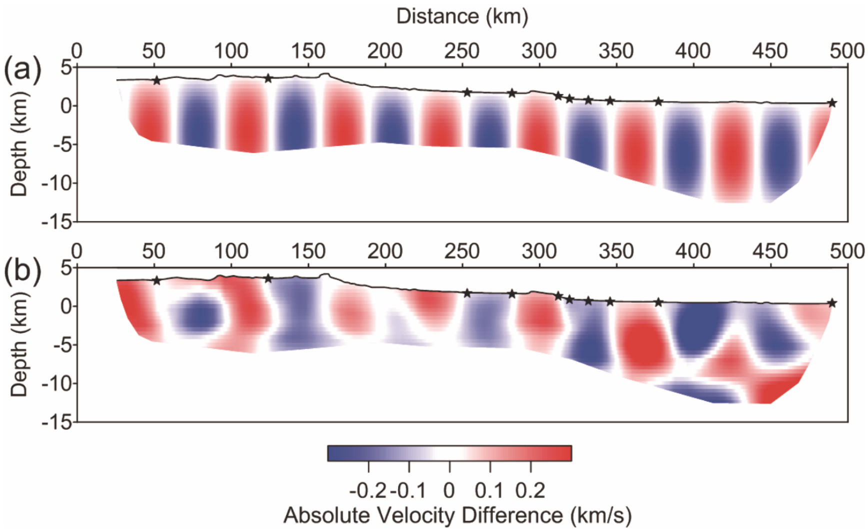

Figure S2. Checkerboard resolution test. The space scale of velocity perturbation is 30 × 15 km, and the value scale is shown at the bottom. Black stars on the surface denote shot positions.

The second resolution analysis is a restoring resolution test. In this test, we add a random Gaussian noise with a standard deviation of $\sigma = 0.2$ s to the synthetic data. The result, as shown in Fig. S3, illustrates the same zoning characteristics as with the tomographic result. It suggests that the main features of the tomographic imaging (Fig. 3a) are reliable. In fact, more specific features of upper crust are disclosed by our model, and thus more geodynamic information is revealed from our tomographic result.

S3. Comparison to the model expansion method

In this paper, we use the topography-dependent traveltime tomography (TDTT) scheme[1,2]. It is in contrast to a model expansion scheme[3] which uses the stair-steps approximate of the irregular surface and covers the top with a low-velocity layer that has an artificially-flat plane surface. However, this conventional scheme would cause not only accuracy loss but even also image distortion.

Fig. S4a is the velocity model that is obtained by the conventional scheme, which fills the top layer with an artificial medium having a low velocity of 0.5 km/s. In general, this model and that obtained from TDTT (Fig. 3a) have a similar main feature, and a small difference between them. We have seen uniformly distributed ray paths in the TDTT model, a key element for TDTT to have a high resolution. But, in the result obtained by

using the conventional method, there are messy rays and false rays above the surface, due to low-velocity filling (Fig. S4b).

In the Songpan-Ganzi terrane, the undulating sedimentary interface has not been clearly imaged, which may result from false rays shooting across the surface to the low-velocity filling. Moreover, the high-velocity anomalies (6.3 km/s) that are distributed in the depth range of 5–10 km may be artefacts that are also caused by erroneous ray distribution, which cannot be interpreted geologically.

Within the Sichuan basin, the velocity contours are more chaotic in the conventional result, which is inconsistent with the stable sedimentary environment of the Sichuan basin. Moreover, no compression-torsion can be interpreted from the velocity structure that was obtained by the conventional Hole's method.

In the LMS fault zone, the uplifted basement cannot be clearly observed in this segment of profile. There is only a high velocity anomaly that is distributed beneath the LMS fault belt, with no outcrop on the surface, which is inconsistent with the severe denudation and the Pengguan complex that is distributed on the surface.

In summary, the velocity model that was reconstructed with the TDTT method has a clear and high-resolution image, and discloses significant features for each tectonic block, differing from the result obtained by the Hole's method.

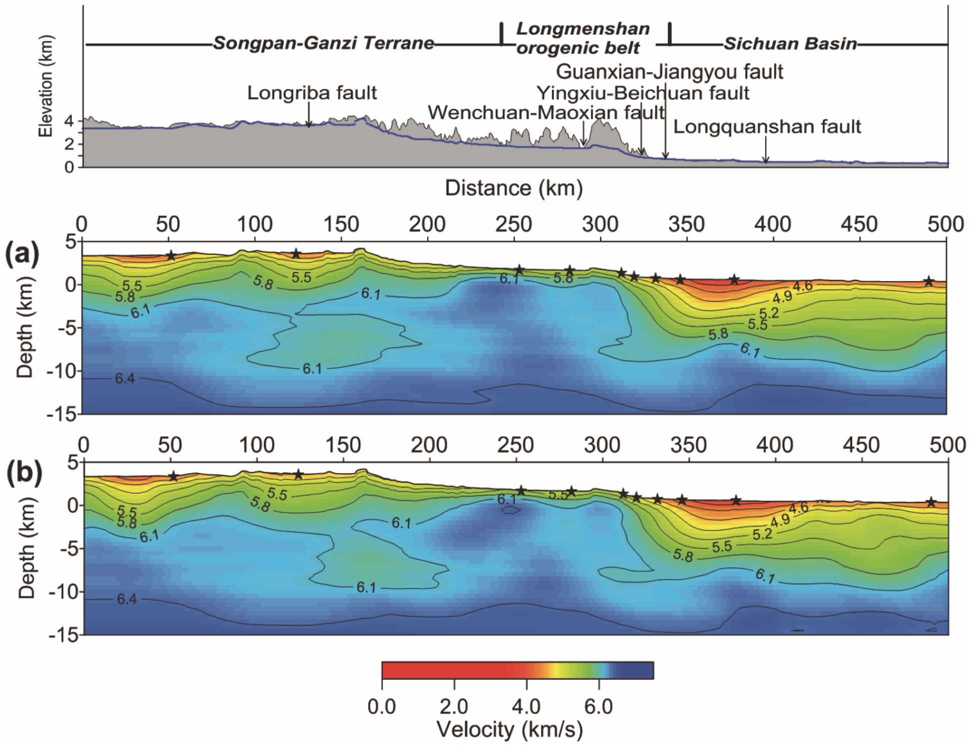

Figure S3. Restoring resolution test. (a) The final inversed model. (b) Restoring resolution test by adding random Gaussian noise (σ = 0.2 s) to the synthetic data from the final inverted model (a), and reconstructed using the TDTT solver.

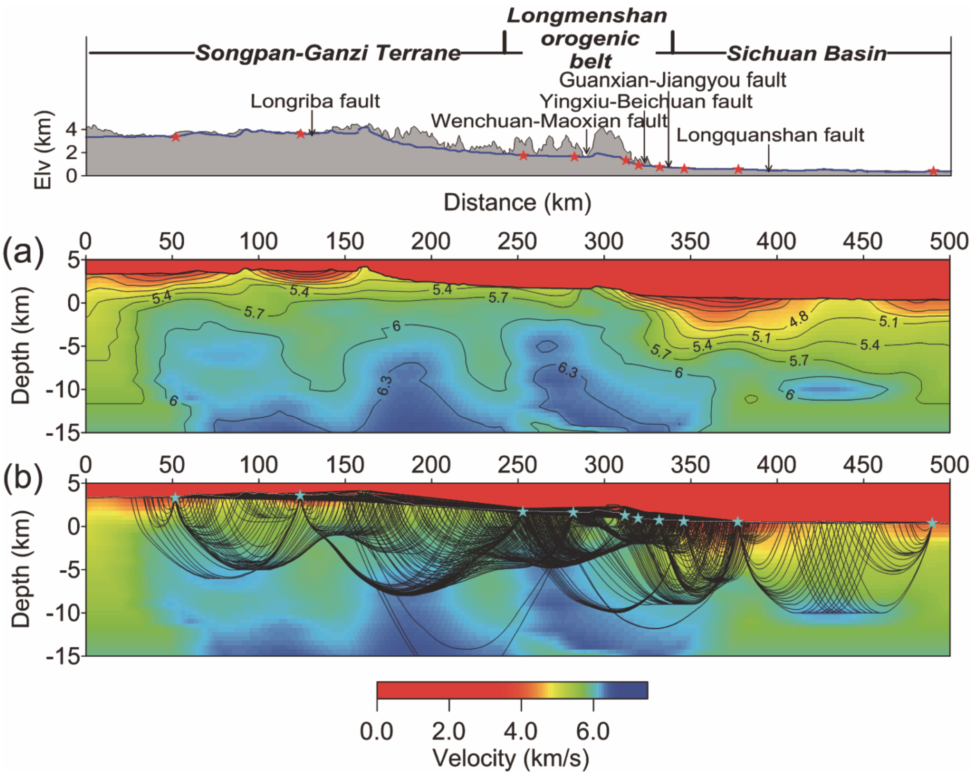

Figure S4. (a) Tomography result of upper crust along the wide-angle seismic profile, calculated according to Hole's scheme[3]. (b) Ray paths passing through each cell, using the conventional Hole's scheme.

S4. Joint inversion of the first-arrival times and a reflection times

In order to verify this upper crustal structure model, we have conducted a joint inversion of the first-arrival times and reflection times. We picked a reflection phase on the seismic records, which presumably is reflected from the intra-crustal interface. This reflection phase is labelled as P2, as shown in Fig. S5 for shot no. 2 and 6.

We have implemented the joint inversion of the first arrival (Pg) and the reflected arrival (P2) times[4]. A grid size of 501×26 with a horizontal and vertical spacing of 1 km was employed in order to parameterise the model. In the joint inversion, the initial model was constructed based on the upper crustal model that was obtained by the Pg arrival time inversion (Fig. 3a). Fig. 4 (in main text) is the upper crustal structure that was obtained by this joint inversion. The final root-mean-square residual is reduced to 0.12 s and 0.16 s, for Pg and P2 respectively, suggesting a satisfactory convergence level.

The averaged depth of the reflection interface is approximately 17 km. The updated velocity model confirms that the high-velocity anomaly is reliable at a depth greater than 5 km beneath the Longmenshan faults. More importantly, it supports the crustal-scale interpretive model.

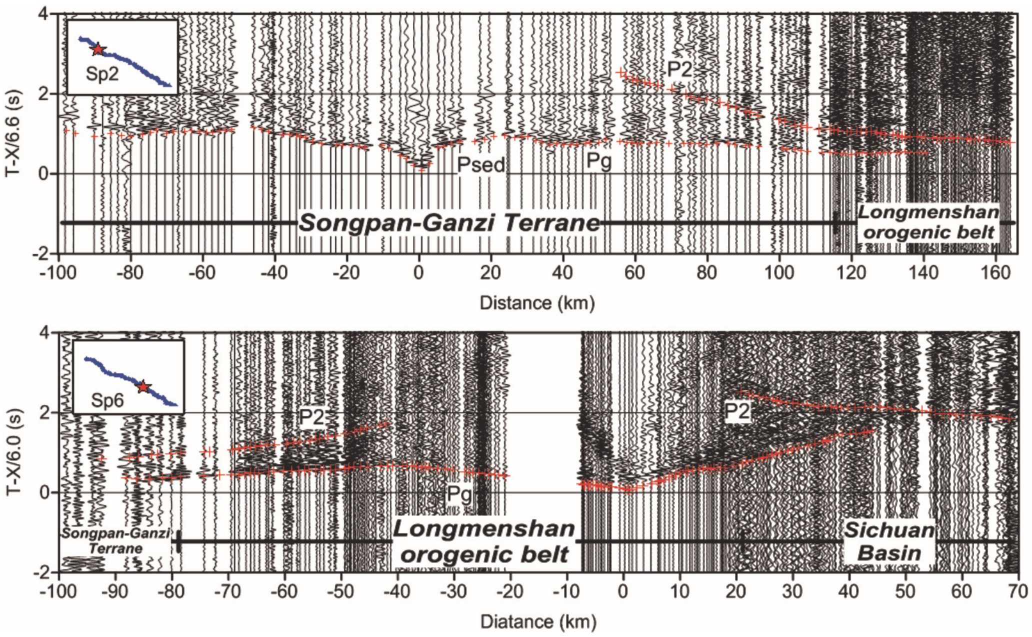

Figure S5. Picked Pg and P2 phases along the seismic records of shot no. 2 and 6, respectively.

References

1. Lan, H. & Zhang, Z. Topography-dependent eikonal equation and its solver for calculating first-arrival traveltimes with an irregular surface. *Geophysical Journal International* **193**, 1010–1026 (2013).
2. Ma, T. & Zhang Z. Calculating ray paths for first-arrival travel times using a topography-dependent eikonal equation solver. *Bulletin of the Seismological Society of America* **104**, 1501–1517 (2014).
3. Hole, J. Nonlinear high-resolution three-dimensional seismic travel time tomography. *Journal of Geophysical Research* **97**, 6553–6562 (1992).
4. Hobro, J. Singh, S. & Minshull, T. Three-dimensional tomographic inversion of combined reflection and refraction seismic traveltime data. *Geophysical Journal International* **152**, 79–93 (2003).
